# Medical thoracoscopy/pleuroscopy: is it underutilized?

**DOI:** 10.1080/20018525.2016.1270078

**Published:** 2017-01-06

**Authors:** Deniz Koksal, Sevinc Sarinc Ulasli, Salih Emri

**Affiliations:** ^a^ Department of Chest Diseases, Hacettepe University School of Medicine, Ankara, Turkey

Thoracoscopy is a technique introduced more than 100 years ago. The first reported thoracoscopy is thought to be performed as early as 1866 by an Irish urologist Francis Richard Cruise. He examined the pleural cavity through pleurocutenous fistula of an 11-year-old girl with empyema.[1] However, Hans-Christian Jacobaeus, an internist from Sweden, is regarded to be the father of thoracoscopy, since he introduced the technique together with laparoscopy in 1910 and made it known worldwide by many publications and lectures.[2] Jacobaeus used thoracoscopy for the diagnosis of tuberculous effusions and lysis of adhesions due to tuberculosis in the pre-antibiotic era of tuberculosis treatment. Jacobaeus operations were applied nearly for 40 years worldwide almost exclusively for this purpose. Between 1950 and 1970, the usage of anti-tuberculosis drugs and a diagnostic yield of 70% with closed pleural needle biopsies decreased the need for thoracoscopy. During the following 20 years (1970–1990), tuberculosis decreased whereas malignancies increased, causing thoracoscopy to become popular again. In 1991, getting inspiration from the experience of abdominal surgeons on minimally invasive surgery, thoracic surgeons introduced ‘surgical thoracoscopy’ or ‘video-assisted thoracic surgery’ (VATS).[3]

In 1994, the term ‘medical thoracoscopy’ (MT) was introduced by pulmonologists to better differentiate thoracoscopies performed by interventional pulmonologists and chest surgeons.[4] Some authors support the usage of old term ‘pleuroscopy’ (P) which allows an easier distinction. After the introduction of the semi-rigid pleuroscope by Olympus Corporation, Tokyo, Japan, the term P became more popular and today both terms are used interchangeably.[5] MT/P is a technique performed by chest physicians in a clean endoscopy suite. It is less invasive than VATS, since it can be performed under local anesthesia and conscious sedation through a single entry. However, VATS is performed by chest surgeons in an operating room, under general anesthesia, selective tube intubation and with multiple entries. VATS is more invasive and more expensive.[3]

The main indications of MT/P are diagnosis of undiagnosed exudative pleural effusions and talc pleurodesis of malignant pleural effusions. Currently it is the second most important endoscopic technique in pulmonary medicine after bronchoscopy and considered as an integral part of interventional pulmonology. It is easier to learn thoracoscopy than flexible bronchoscopy if there is sufficient expertise in thoracenthesis and chest tube replacement.[2] Although varying from region to region, the numbers of chest physicians performing MT/P are slightly increasing.

The present paper focuses on documenting publications related to MT/P. Its aim is to provide an overview of the nature of publications with regard to the years of publication, publication types, discipline and country of origin. We searched all publications about MT/P published between January 1945 and August 2016, and documented the characteristics of the publications regarding publication types, discipline and country of origin by using Thomson Reuters Web of Knowledge Web of Science software (Thomson Reuters Corporation, New York, NY, USA). Web of Science is the largest abstract and citation database of peer-reviewed literature: scientific journals, books, and conference proceedings (http://apps.webofknowledge.com/). ‘Medical Thoracoscopy’ or ‘Pleuroscopy’ were selected as ‘topic’ and ‘all years to present (August, 2016)’ was selected as ‘date range.’ A total of 622 documents were retrieved.

**Figure 1.**
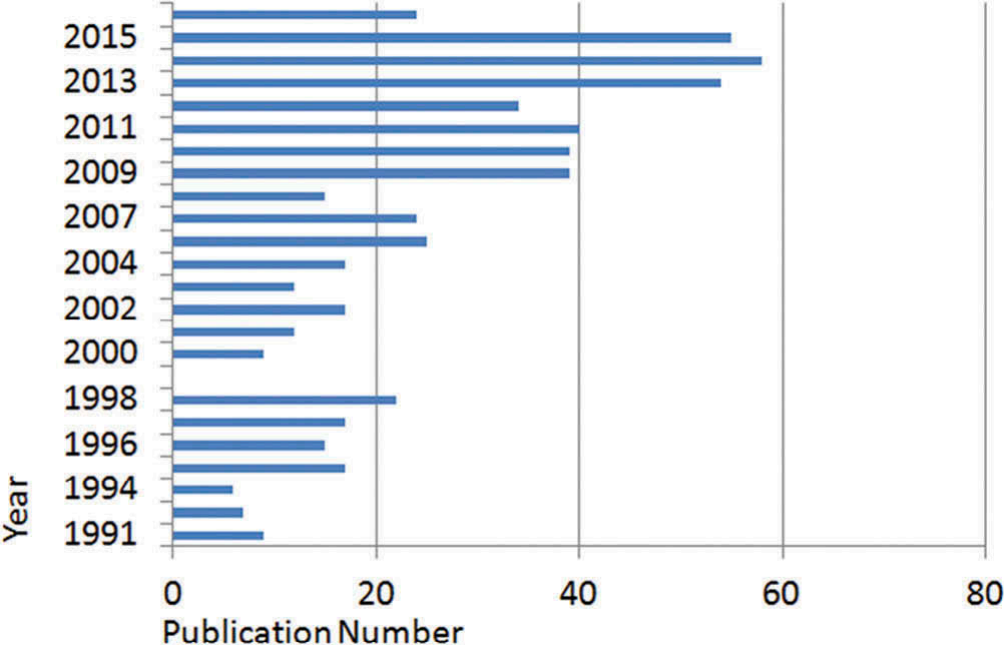
Distribution of publications related to MT/P by year of publication between 1991 and 2016.

Most of the documents (*n* = 584) were published in 1991–2016, a period in which MT/P was popular again 80 years after the Jacobaeus operations. The distribution of publications among years between 1991 and 2016 is demonstrated in Figure 1. In the last decade there was an increase in the number of publications; the highest number was 58 in 2014. The types and the distributions of publications according to disciplines are depicted in Tables 1 and 2, respectively. The most common type of publication was research articles (*n* = 425), indicating that the area offers scope for productive research. As expected, the respiratory system was the most common discipline studied in MT/P.

**Table 1.** Distribution of publication types on MT/P.

Publication types	Number of publications
Research article	425
Review	69
Abstract	76
Conference paper	48
Letter	19
Editorial	14
Note	5
Other	1

**Table 2.** Distributions of publications according to disciplines (top 5 disciplines).

Rank	Research area	Number of publications
1	Respiratory system	368
2	Surgery	146
3	General internal medicine	139
4	Cardiovascular system	74
5	Oncology	53

The 15 countries which publish the most scientific research into MT/P are given in Table 3. The highest numbers of publications were from USA (*n* = 161), UK (*n* = 69) and France (*n* = 62). Re-evaluating the number of publications by region, the highest number of publications were from Europe (*n* = 269), USA (*n* = 161) and Eastern countries (*n* = 143), respectively. Although the highest number on a country basis is from USA, by region the highest number of publications is from Europe; thoracoscopy began to recover in the 1970s in continental Europe where some pulmonologists became expert in this interventional technique.[5] In several European countries, MT/P has been a part of training programs in respiratory medicine for many years and it became popular later in the USA.[3] Our data reflect this situation.

**Table 3.** Global distribution of scientific publications about MT/P (top 15 countries).

Rank	Country	Number of publications
1	USA	161
2	UK	69
3	France	62
4	China	41
5	Japan	35
6	Italy	31
7	Switzerland	25
8	Greece	22
9	Germany	22
10	Spain	19
11	Belgium	19
12	Taiwan	18
13	Singapore	18
14	India	16
15	Israel	15

In conclusion; our data revealed that despite being a very old technique, the number of publications about MT/P is very limited, but has tended to increase in the last decade. A generation of physicians is already familiar with this interventional technique; their teams use MT/P for diagnostic and therapeutic evaluation of many pulmonary diseases and their experience is passed on to the next generation. However, it is difficult to start from scratch when performing MT/P. In our opinion there are three main reasons for this: the lack of a dedicated chest physician, the lack of equipment and clean endoscopy units, and the lack of support from other disciplines such as anesthesia and chest surgery. Therefore, many patients are referred to chest surgeons for VATS and faced with a more invasive and costly procedure.
